# The Society for Cardiovascular Magnetic Resonance Registry at 150,000

**DOI:** 10.1016/j.jocmr.2024.101055

**Published:** 2024-07-04

**Authors:** Matthew S. Tong, Jeremy A. Slivnick, Behzad Sharif, Han W. Kim, Alistair A. Young, Lilia M. Sierra-Galan, Kanae Mukai, Afshin Farzaneh-Far, Sadeer Al-Kindi, Angel T. Chan, George Dibu, Michael D. Elliott, Vanessa M. Ferreira, John Grizzard, Sebastian Kelle, Simon Lee, Maan Malahfji, Steffen E. Petersen, Venkateshwar Polsani, Olga H. Toro-Salazar, Kamran A. Shaikh, Chetan Shenoy, Monvadi B. Srichai, Jadranka Stojanovska, Qian Tao, Janet Wei, Jonathan W. Weinsaft, W. Benjamin Wince, Priya D. Chudgar, Matthew Judd, Robert M. Judd, Dipan J. Shah, Orlando P. Simonetti

**Affiliations:** a Division of Cardiovascular Medicine, Department of Internal Medicine, The Ohio State University, Columbus, Ohio, USA; b Division of Cardiovascular Medicine, The University of Chicago Medicine, Chicago, Illinois, USA; c Krannert Cardiovascular Research Center, Indiana University School of Medicine, Indianapolis, Indiana, USA; d Division of Cardiology, Department of Medicine, Duke University, Durham, North Carolina, USA; e Department of Anatomy and Medical Imaging, University of Auckland, Auckland, New Zealand; f School of Biomedical Engineering and Imaging Sciences, King's College London, London, United Kingdom; g Cardiology Department of the Cardiovascular Division of The American British Cowdray Medical Center, Mexico City, Mexico; h Ryan Ranch Center for Advanced Diagnostic Imaging, Salinas Valley Health, Salinas, California, USA; i Division of Cardiology, University of Illinois at Chicago, Chicago, Illinois, USA; j Harrington Heart and Vascular Institute, University Hospitals and Case Western Reserve University, Cleveland, Ohio, USA; k Department of Medicine, Memorial Sloan Kettering Cancer Center, New York, New York, USA; l Ascension St. Vincent's Medical Center, Jacksonville, Florida, USA; m Sanger Heart & Vascular Institute, Atrium Health, Charlotte, North Carolina, USA; n Oxford Centre for Clinical Magnetic Resonance Research (OCMR), Division of Cardiovascular Medicine, Radcliffe Department of Medicine, John Radcliffe Hospital, University of Oxford, Oxford, United Kingdom; o Department of Radiology, Virginia Commonwealth University, Richmond, Virginia, USA; p Deutsches Herzzentrum der Charité, Department of Cardiology, Angiology and Intensive Care Medicine, Campus Virchow Clinic, Berlin, Germany; q German Centre for Cardiovascular Research, Berlin, Germany; r Heart Center, Ann & Robert H. Lurie Children's Hospital of Chicago, Chicago, Illinois, USA; s Houston Methodist DeBakey Heart and Vascular Center, Houston, Texas, USA; t William Harvey Research Centre, Queen Mary University London, London, United Kingdom; u Barts Heart Centre, St Bartholomew’s Hospital, Barts Health National Health Service Trust, London, United Kingdom; v Piedmont Heart Institute, Piedmont Atlanta Hospital, Atlanta, Georgia, USA; w Pediatric Cardiology, Connecticut Children's Medical Center, University of Connecticut School of Medicine, Hartford, Connecticut, USA; x Seton Heart Institute, Seton Medical Center, Kyle, Texas, USA; y Cardiovascular Division, Department of Medicine, University of Minnesota Medical School, Minneapolis, Minnesota, USA; z Departments of Cardiology and Radiology, Georgetown University School of Medicine, Washington, District of Columbia, USA; aa Department of Radiology, Langone Health, New York University, New York, New York, USA; ab Department of Imaging Physics, Delft University of Technology, Delft, the Netherlands; ac Smidt Heart Institute, Cedars-Sinai Medical Center, Los Angeles, California, USA; ad Division of Cardiology, Department of Medicine, Weill Cornell Medicine – New York Presbyterian Hospital, New York, New York, USA; ae St. Vincent Heart Center of Indiana, Indianapolis, Indiana, USA; af Department of Radiology, Jupiter Hospital, Mumbai, Maharashtra, India; ag Heart Imaging Technologies, LLC, Durham, North Carolina, USA; ah Department of Radiology, The Ohio State University, Columbus, Ohio, USA

**Keywords:** Cardiovascular magnetic resonance, Late gadolinium enhancement, Infarction, Registry, Real-world evidence

## Abstract

**Background:**

Cardiovascular magnetic resonance (CMR) is increasingly utilized to evaluate expanding cardiovascular conditions. The Society for Cardiovascular Magnetic Resonance (SCMR) Registry is a central repository for real-world clinical data to support cardiovascular research, including those relating to outcomes, quality improvement, and machine learning. The SCMR Registry is built on a regulatory-compliant, cloud-based infrastructure that houses searchable content and Digital Imaging and Communications in Medicine images. The goal of this study is to summarize the status of the SCMR Registry at 150,000 exams.

**Methods:**

The processes for data security, data submission, and research access are outlined. We interrogated the Registry and presented a summary of its contents.

**Results:**

Data were compiled from 154,458 CMR scans across 20 United States sites, containing 299,622,066 total images (∼100 terabytes of storage). Across reported values, the human subjects had an average age of 58 years (range 1 month to >90 years old), were 44% (63,070/145,275) female, 72% (69,766/98,008) Caucasian, and had a mortality rate of 8% (9,962/132,979). The most common indication was cardiomyopathy (35,369/131,581, 27%), and most frequently used current procedural terminology code was 75561 (57,195/162,901, 35%). Macrocyclic gadolinium-based contrast agents represented 89% (83,089/93,884) of contrast utilization after 2015. Short-axis cines were performed in 99% (76,859/77,871) of tagged scans, short-axis late gadolinium enhancement (LGE) in 66% (51,591/77,871), and stress perfusion sequences in 30% (23,241/77,871). Mortality data demonstrated increased mortality in patients with left ventricular ejection fraction <35%, the presence of wall motion abnormalities, stress perfusion defects, and infarct LGE, compared to those without these markers. There were 456,678 patient-years of all-cause mortality follow-up, with a median follow-up time of 3.6 years.

**Conclusion:**

The vision of the SCMR Registry is to promote evidence-based utilization of CMR through a collaborative effort by providing a web mechanism for centers to securely upload de-identified data and images for research, education, and quality control. The Registry quantifies changing practice over time and supports large-scale real-world multicenter observational studies of prognostic utility.

## Graphical abstract

**Figure:**
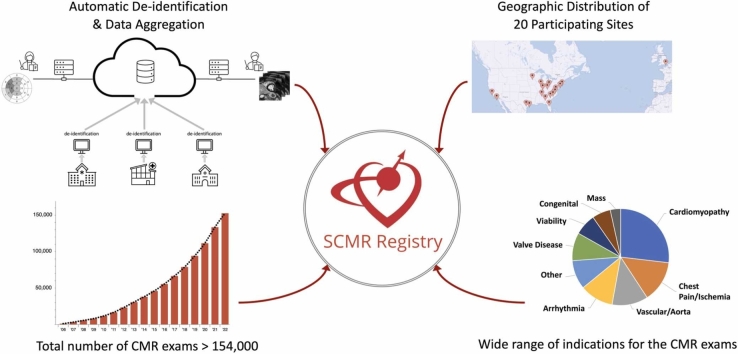
Central illustration: Composite diagram representation of the SCMR Registry, from automated data de-identification and aggregation (top left), cumulative growth over 5 years since creation (bottom left), geographic locations of participating sites (top right), and distribution of CMR indications (bottom right).

## 1 Introduction

Cardiovascular magnetic resonance (CMR) has emerged over the past 20 years as the advanced imaging modality of choice for diagnosing structural heart disease, ischemic heart disease, myopericarditis, and cardiomyopathies [1], [2], [3], [4], [5]. At the same time, data on practice and utilization trends for CMR in the United States are typically limited to the Medicare population (ages 65 and above) or randomized clinical trials in academic centers [6].

Without a mechanism to track real-world clinical data points across all age groups (including those under 65 years) and settings, quantifying the utilization rate and identifying barriers to appropriate guideline-based adoption of CMR remain a challenge. An observational multicenter registry allows efficient large-scale analysis of outcomes and cost-effectiveness in community-based settings as well as academic centers; such data cannot be generated with prospective clinical trials alone [7]. Randomized controlled trials (RCTs), while narrowly focused on specific populations, can be complemented by real-world evidence (RWE) studies across multiple subsets, with higher numbers of outcomes for prognostication. As a result, a large registry provides the ideal framework for studies in implementation science, including national quality assurance and machine learning (ML) initiatives, while providing educational value for practicing physicians.

The Society for Cardiovascular Magnetic Resonance (SCMR) Registry was initiated in 2014 as the Global CMR (GCMR) registry; under the leadership of Dr. Raymond Y. Kwong, it enrolled 21 international sites, contributing over 62,000 CMR exams by 2016 [8]. One of the successes of the GCMR registry was translated through the SPINS (Stress CMR Perfusion Imaging in the United States: A Society for Cardiovascular Resonance Registry Study) trial, showing a significant reduction in downstream costs and major adverse cardiac events (MACE) in the setting of a normal stress CMR [9]. This and other CMR registry publications have demonstrated the value of CMR across both ischemic and non-ischemic cardiovascular diseases [8], [9], [10], [11], [12], [13], [14], [15], [16], [17], [18], [19], [20], [21], [22], [23], [24], [25], [26]. A number of SCMR Registry-based research studies to date are summarized in Table 1.

**Table 1:** Summary of previous SCMR Registry publications.

Study	Date	Study design	n	Sites	National/international	Images
Kwong et al., GCMR [8]	2017	Registry design	62,456	17	International, intercontinental	N
Romano et al., CloudCMR* [15]	2018	MAPSE and outcomes in HTN	1735	4	National	Y
Kwong et al., SPINS [9]	2019	Stress CMR outcomes	2349	13	National	N/A
Heitner et al., CloudCMR* [14]	2019	Stress CMR mortality	9151	7	National	Y
Antiochos et al., SPINS [10]	2020	Stress CMR net reclassification	1698	13	National	N/A
Antiochos et al., SPINS [11]	2020	Unrecognized MI outcomes	2349	13	National	N/A
Ge et al., SPINS [12]	2020	Stress CMR cost-effectiveness	2349	13	National	N/A
Ge et al., SPINS [13]	2020	Stress CMR outcomes LVEF <50%	582	13	National	N/A
Ge et al., SPINS [22]	2021	Stress CMR obesity performance	1177	13	National	N/A
Roifman et al., GCMR/SCMR [19]	2022	CMR and heart failure	6654	13	International, intercontinental	N
Kochav et al., SCMR [20]	2022	CMR and ischemic mitral regurgitation	2647	7	National	Y
Vidula et al., SCMR [21]	2022	CMR and COVID-19	1047	18	International, intercontinental	N
Antiochos et al., SPINS [23]	2022	Stress CMR outcomes in known CAD	755	13	National	N/A
Moschetti et al., EuroCMR + SPINS [25]	2022	Stress CMR cost-effectiveness	59,996	72	International, intercontinental	N
Malahfji et al., SCMR [26]	2023	CMR and aortic regurgitation	458	4	National	N
Heydari et al., SPINS [24]	2023	Stress CMR sex-specific performance	2349	13	National	N

*CAD* coronary artery disease, *CMR* cardiovascular magnetic resonance, *COVID-19* coronavirus disease 2019, *EuroCMR* European CMR Registry, *GCMR* Global CMR Registry, *HTN* hypertension, *MAPSE* mitral annular plane systolic excursion, *LVEF* left ventricular ejection fraction, *SCMR* Society for Cardiovascular Magnetic Resonance, *SPINS* Stress Perfusion Imaging in the United States.

*CloudCMR early iteration of SCMR.

One key component missing from previous CMR registries has been the availability of Digital Imaging and Communications in Medicine (DICOM) images. Building on the success of the GCMR registry and the SPINS trial, in 2018, the SCMR sought to expand the capabilities of the Registry to include DICOM image data and to provide worldwide database searching capabilities. Following the formal evaluation of proposals from multiple organizations, the SCMR selected Heart Imaging Technologies, LLC (Raleigh, North Carolina), a subsidiary of Intelerad Medical Systems Incorporated (Montreal, Quebec, Canada), as its partner to expand the scope and functionality of the Registry. The SCMR Registry now includes the infrastructure for a centralized, cloud-based database that is compliant with the Health Insurance Portability and Accountability Act (HIPAA). Under the SCMR mission, “Improving global cardiovascular health by leveraging the advantages of CMR,” the Registry serves to promote a collaborative global effort to support evidence-based CMR utilization. The SCMR Registry provides several important, unique features: worldwide access to the Registry database through a web-based portal, direct access to DICOM image data, and tracking of all-cause mortality. This accessibility and outcome data translate into higher impact research opportunities and health care provider education to enhance cardiovascular health.

In this manuscript, the processes for data security, data submission, and research access from initiation to project implementation are outlined. With the Registry at over 150,000 CMR studies, we present a summary of its contents.

## 2 Methods

### 2.1 Vision of the SCMR Registry

The SCMR Registry supports the SCMR mission through the following objectives:

• Promote evidence-based utilization of CMR through a collaborative global effort.
• Provide a web mechanism for CMR centers to upload de-identified patient data, CMR indications, and images that incorporate state-of-the-art data security and privacy standards.
• Provide a mechanism for tracking patient outcomes (death, other clinical events).
• Support global access to make registry data available to the wider CMR research community.

### 2.2 Data security

**Fig. 1:**
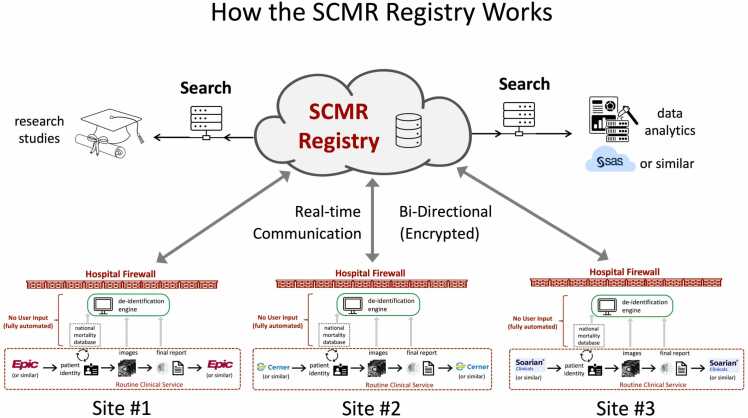
Diagram process of the automated de-identification and cloud aggregation of clinical data into the SCMR Registry platform. *SCMR* Society for Cardiovascular Magnetic Resonance.

The SCMR Registry is built on the HeartIT CloudCMR service. Development, testing, and production use of the CloudCMR software were funded in part by a series of Small Business Technology Transfer grants from the U.S. National Institutes of Health (NIH) (R42HL080843, R42HL106864, and R42HL117397). CloudCMR provides a regulatory-compliant, cloud-based infrastructure with easily accessible and searchable content. CloudCMR is currently hosted by Amazon Web Services. Intelerad’s security policies are regularly audited by a registrar—B.S.I.—to certify compliance with the ISO 27001:2013 standard and are also System and Organization Controls (SOC) 2 type II certified. SOC 2 is an industry-standard that provides detailed information and assurance about the controls at a service organization relevant to the security, availability, and processing integrity of the systems used to process user data, and the confidentiality and privacy of the information processed by these systems. The process of de-identification and cloud aggregation of clinical data is fully automated (Fig. 1).

The SCMR Registry platform ensures that the cloud data are uploaded in such a manner that patients cannot be identified, directly or through identifiers linked to the patients. Specifically, the software within each hospital firewall securely maintains patient identifiers and private health information, but these data are never transmitted to the cloud database. A forward-only association is maintained between the local and cloud datasets that allow the cloud data to be continually updated with new local information, e.g., subsequent patient mortality, without the need to maintain patient identity within the Registry. This structure prevents researchers from identifying patients based on cloud data, even if that data originated from their own institution. This unique software architecture is specifically designed to allow for the addition and updating of new information locally, such as a patient’s death 2 years after a magnetic resonance imaging scan, without violating patient privacy. This forward-only association between the local and cloud data is not accessible to any individual but only exists within the software that controls the communication between the local and cloud systems. The platform maintains ongoing updates to security protocols and compliance standards, ensuring alignment with international privacy laws and providing transparency through regular security audit reports.

### 2.3 SCMR Registry participation

To enroll as a participating site in the Registry, each site must follow a multi-step process that involves local institutional leadership, the local Institutional Review Board (IRB), and Information Technology (IT) support. The SCMR Registry Participation Agreement document governing the submission and utilization of data is signed as a legal agreement by both the SCMR and the participating site. While the submission of de-identified data to the Registry is not considered human subjects research in the United States according to Health and Human Services guidelines, the local IRB typically reviews the participation terms and de-identification process and makes this determination. Contribution of data from outside the United States must be compliant with local institutional and national privacy laws. A data security review is generally required, and this is performed by the local IT department in collaboration with HeartIT. Once these tasks are completed, a Registry Connector System is installed to extract, de-identify, and upload images and data to the Registry from the existing picture archiving and communication system and CMR reporting systems. There is an initial charge for installation and an annual maintenance fee for the Registry Connector System.

### 2.4 Data query and access for study design

Once a site is connected, de-identified images and finalized CMR reports from consecutive scans are uploaded to the Registry daily. All data are submitted in accordance with HIPAA and other privacy legislation depending on the country of origin. Registry data remain in the control of the participating center, and the decision is made by the CMR medical director at each site whether to allow or restrict data access on a project-specific basis.

Prospective study investigators at participating sites can query the Registry independently, but investigators from non-contributing institutions must collaborate with a participating site to access and search the Registry. This collaboration provides insight into available Registry datasets, other participating sites, and potential research limitations. Data queries are performed on the Registry website through a set of conditional statements of available data elements to meet the inclusion and exclusion criteria for the proposed study (e.g., review of all CMR scans with left ventricular ejection fraction [LVEF] less than 50% and more than mild mitral regurgitation). The investigator then applies for data access, describing the project and the data available, and specifying which participating sites will be invited to participate. Committee approval does not guarantee access; this decision remains with each individual participating site. Investigators are also encouraged to review the list of active projects on the SCMR Registry website (https://scmr.org/page/Registry) to minimize redundancy. The SCMR Committee follows a proposal review process similar to an NIH grant review, scoring each proposal based on alignment with the SCMR Mission, the potential impact on the field, feasibility based on the availability of data and required effort, and the strength of the investigators. This process ensures alignment with the SCMR vision and that the necessary capabilities and resources are in place to complete the project. The details of the review process and scoring criteria are posted on the SCMR Registry website (https://scmr.org/wp-content/uploads/2023/12/6.21_registry_data_access_re.pdf, https://scmr.org/wp-content/uploads/2023/12/Registry_Data_Access_App_Rev.pdf). The SCMR Registry Committee reviews submissions quarterly, with subsequent coordination with a Committee representative and investigator upon approval or rejection. The SCMR currently does not impose monetary charges to the prospective investigator associated with querying, accessing the Registry data, or submitting for project approval.

**Fig. 2:**
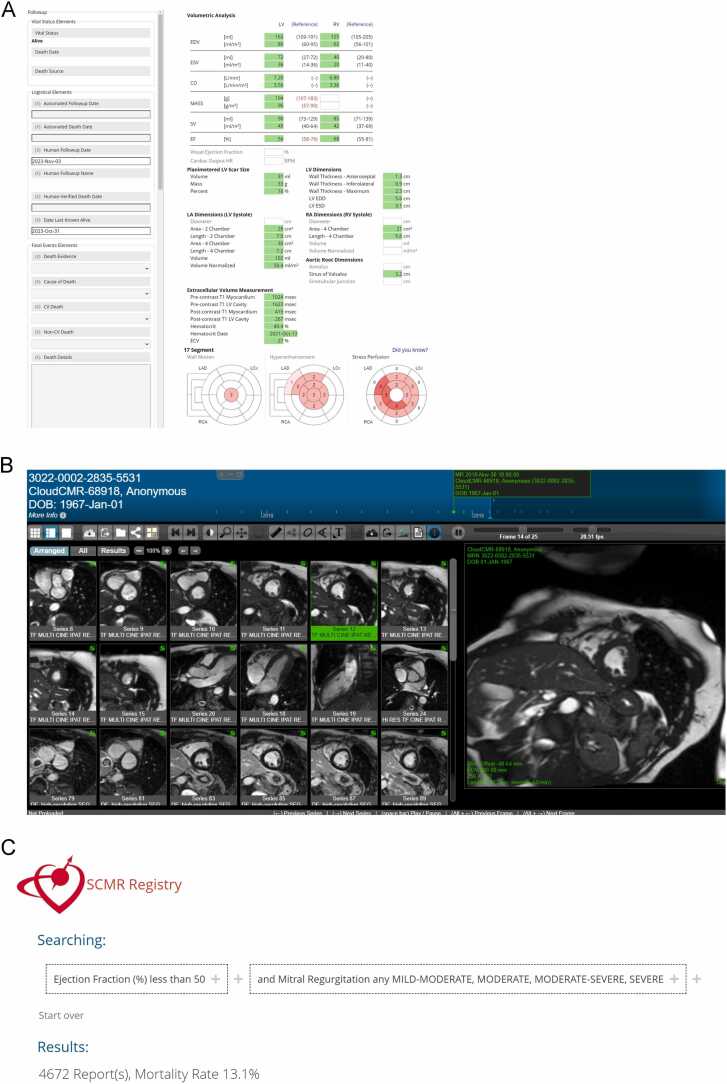
Example of a de-identified report (A), respective DICOM images (B), and query interface (C) in the SCMR Registry. *CO* Cardiac output, *EDD* End-diastolic diameter, *EDV* End-diastolic volume, *EF* Ejection fraction, *ESD* End-systolic diameter, *ESV* End-systolic volume, *DICOM* Digital Imaging and Communications in Medicine, *LAD* left anterior descending artery, *LCX* left circumflex artery, *LV* left ventricle, *RA* Right atrium, *RCA* right coronary artery, *RV* Right ventricle, *SCMR* Society for Cardiovascular Magnetic Resonance.

Once the project is approved with engagement from a sufficient number of site investigators, the relevant data, including DICOM images, are aggregated into an SCMR Registry project folder that can be accessed only by those investigators involved in the project, and for the purpose of the project only. The sharing of de-identified data and DICOM images is at the discretion of each participating site, meaning that investigators of registry-approved projects may only access data that have been expressly shared by a participating site. An example of a CMR report, image set, and query interface from the investigator's viewpoint is shown in Fig. 2A-C. The data and results are to be used for academic purposes only, and all research results are expected to be made publicly available. Any artificial intelligence (AI) or ML models trained using Registry data and the associated source code must be published and made available publicly as open source without cost or limitation. Bi-monthly meetings are held with the Registry Committee and investigators on progress and support. The Registry Committee also reviews manuscripts before submission to ensure SCMR vision alignment. The data access policy and process are posted on the SCMR Registry website in the SCMR Registry Data Access Policy section (https://scmr.org/wp-content/uploads/2023/12/2021_registry_access_applic.docx).

### 2.5 Study population and data/image analysis

The Registry includes consecutive CMR exams dating back as far as September 2001 (starting dates vary by center) to the present. Each participating site used its own local institutional protocols for patient demographics, indication-specific imaging, and parameter definitions (e.g., race, parameter severity). All anonymized CMR data points presented here were collected in October 2022 according to pre-specified fields in the HeartIT imaging report as detailed above, including patient demographics, history, medications, indications, United States-specific procedural codes, mortality, and CMR findings. De-identified CMR images—including cine imaging, tissue mapping, perfusion imaging, late gadolinium enhancement (LGE), and phase contrast imaging—can be viewed within the viewing platform. Defined searchable CMR fields include chamber and vessel sizes and function, valve morphology with qualitative/quantitative function, stress and non-stress perfusion findings, and tissue characterization, such as LGE segmentation (Fig. 2A and B). The Supplemental Table shows every available data field that can be recorded and searched within the SCMR Registry.

### 2.6 Statistics

Descriptive statistical analyses were primarily performed to evaluate the contents of the Registry. Continuous variables are expressed as mean ± SD, and median with interquartile range (IQR) for normal and skewed distributions, respectively. Categorical variables are expressed as counts with percentages. Mortality was assessed between those with and without 1) LVEF ≤35%, 2) regional LV wall motion abnormalities, 3) abnormal qualitative stress perfusion, and 4) infarct-pattern LGE using Kaplan-Meier analysis and compared using the Log-Rank test. A p-value <0.05 was used to establish statistical significance. All statistical analyses were performed using SAS JMP v16.2.0 (Cary, North Carolina).

## 3 Results

### 3.1 Site participation and available exams

The SCMR Registry has compiled 154,458 exams across 20 participating sites in the United States. Table 2 shows the current participating sites to date. There is one European site and six other US sites with an active SCMR Registry Participation Agreement that have not yet started contributing data.

**Table 2:** Participating sites within the Society for Cardiovascular Magnetic Resonance Registry.

Participating site
Ascension St. Vincent’s Southside Hospital, Jacksonville, Florida, USA
Atrium Health Sanger Heart and Vascular Institute, Charlotte, North Carolina, USA
Cedars Sinai Medical Center, Los Angeles, California, USA
Cleveland Clinic, Cleveland, Ohio, USA
Connecticut Children's, Hartford, Connecticut, USA
Duke University, Durham, North Carolina, USA
Houston Methodist Hospital, Houston, Texas, USA
Indiana University Health, Indianapolis, Indiana, USA
Kings College London, United Kingdom
MedStar Georgetown University, Washington, District of Columbia, USA
New York Presbyterian Brooklyn Methodist, Brooklyn, New York, USA
Piedmont Heart Institute, Atlanta, Georgia, USA
Salinas Valley Health Medical Center, Salinas, California, USA
Seton Heart Institute, Austin, Texas, USA
St. Vincent Heart Center of Indiana, Indianapolis, Indiana, USA
The Ohio State University, Columbus, Ohio, USA
University Hospitals of Cleveland, Cleveland, Ohio, USA
University of Illinois Chicago, Chicago, Illinois, USA
University of Minnesota, Minneapolis, Minnesota, USA
Vanderbilt University Medical Center, Nashville, Tennessee, USA
Virginia Commonwealth University, Richmond, Virginia, USA

### 3.2 Baseline demographics and data completeness

**Fig. 3:**
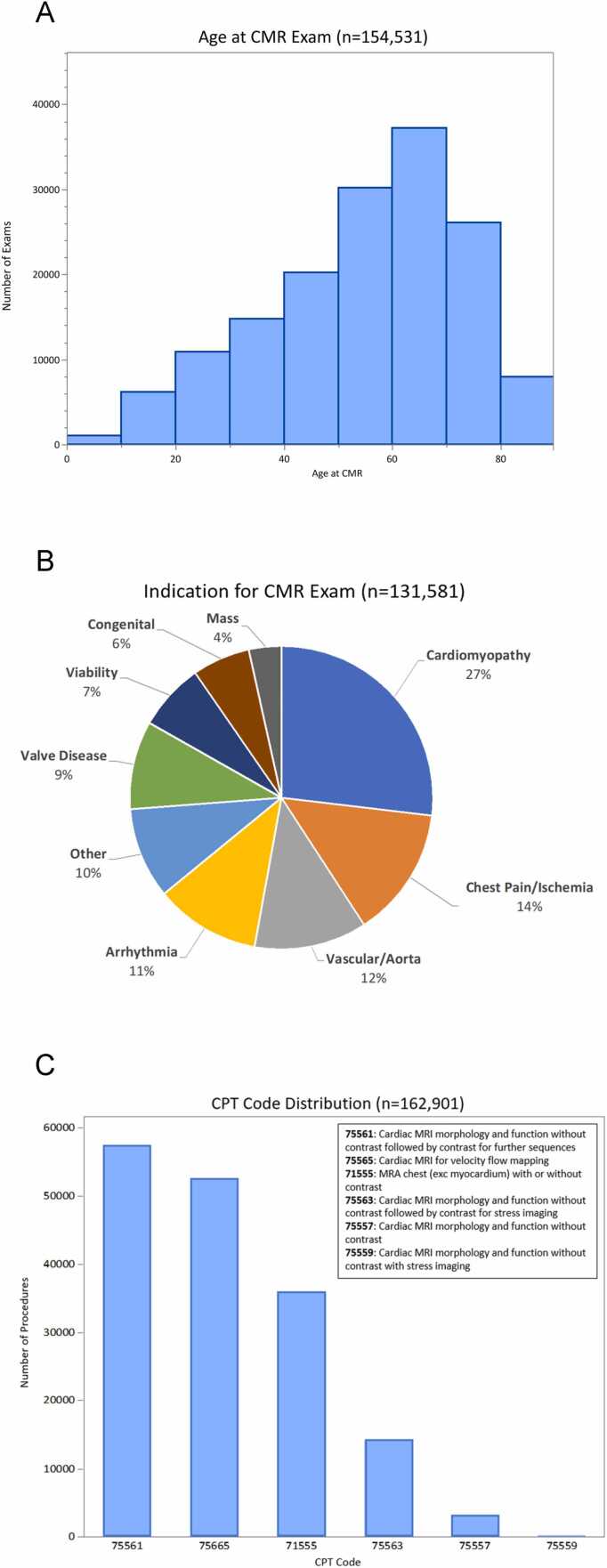
Distribution of CMR exams by age (A), most common CMR indications (B), and distribution of reported CPT codes after the 2008 update. Multiple CPT codes may be reported with each CMR exam (C). *CMR* cardiovascular magnetic resonance, *CPT* current procedural terminology, *MRI* magnetic resonance imaging, *SCMR* Society for Cardiovascular Magnetic Resonance.

**Fig. 4:**
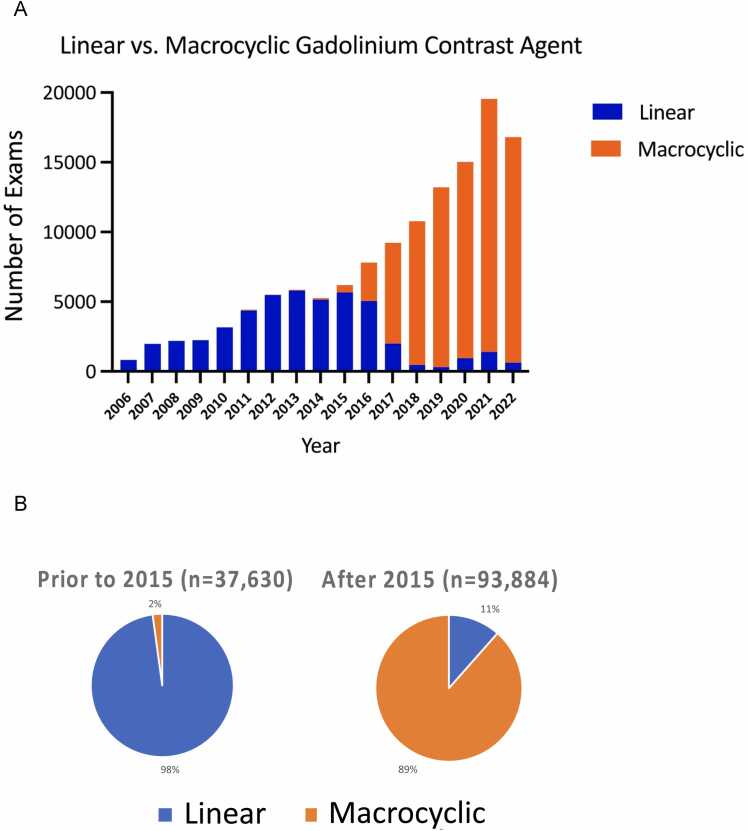
Utilization of linear or macrocyclic gadolinium-based contrast agents (GBCAs) by time (A) and 2015 GBCA designations by the American College of Radiology (B).

Table 3 shows the baseline demographics of patients included in the SCMR Registry and the corresponding completeness for each parameter. These data points originate from structured report fields that are populated by each participating site. The average age was 58 years (minimum age 1 month, maximum age >90 years old); Fig. 3A shows the age distribution of the cohort. Among those reporting sex (145,275/154,458, 94%) and race (98,008/154,458, 63%), 44% (63,070/145,275) were female, 72% (69,766/98,008) were Caucasian, and 18% (17,789/98,008) were African American. The most populated data fields were age, sex, body surface area, and magnetic field strength. The top three indications were cardiomyopathy(35,369/131,581, 27%), chest pain (18,323/131,581, 14%), and arrhythmia (14,801/131,581, 11%) (Fig. 3B). While 6% (8,127/131,581) were reported as congenital heart disease, this may be underestimated and were likely integrated into other indications, such as valve disease (12,369/131,581,9%). Fig. 3C shows CMR current procedural terminology (CPT) codes, with code number 75561 (CMR morphology and function with and without contrast) as the most commonly used code (57,195/162,901,35%), followed by code 75565 (CMR velocity flow mapping) at 32% (52,460/162,901), and code 71555 (magnetic resonance angiography chest with or without contrast) at 22% (35,920/162,901). Fig. 4A shows the history of linear and macrocyclic gadolinium-based contrast agent utilization. While 59% (72,920/123,594) used macrocyclic agents in the entire Registry, its use was demonstratively higher than linear agents after 2015 (89% [83,089/93,884] vs 2% [811/37,630], respectively, Fig. 4B).

**Table 3:** Baseline clinical demographics within the Society for Cardiovascular Magnetic Resonance Registry.

Parameter		Value	Number of exams
*Demographics*			154,458
Age, years		58 (43-69)	154,407
Sex, n (% male)		82,205 (56%)	145,275
Race, n (%)	White/Caucasian	69,766 (72%)	98,008
	Black/AA	17,789 (18%)	
	Asian	2391 (2%)	
	Hispanic/Latino	1977 (2%)	
	Other	6085 (6%)	
Field strength	1.5T	100,294 (74%)	135,610
	3T	35,316 (26%)	
*Medical history*			
Hypertension n (%)		51,403 (58%)	88,198
Hyperlipidemia n (%)		40,993 (47%)	88,088
Diabetes n (%)		17,847 (23%)	87,906
Coronary artery disease n (%)		13,076 (21%)	62,953
Moderate-to-severe valve disease n (%)		15,399 (18%)	85,550
Heart failure n (%)		16,508 (19%)	86,884
Tobacco use (prior or current) n (%)		25,444 (30%)	84,813
Family history of CAD n (%)		25,041 (30%)	87,614
Peripheral arterial disease n (%)		1511 (3%)	54,084
Congenital heart disease n (%)		9028 (10%)	87,258
Non-ischemic cardiomyopathy n (%)		7051 (9%)	86,749
Cardiomyopathy subtype n (%)	Amyloid	142 (3%)	5553
	ARVC	72 (1%)	
	HCM	1492 (27%)	
	Idiopathic DCM	1097 (20%)	
	Sarcoid	184 (3%)	
	Other	764 (14%)	
	Unknown	1802 (32%)	
History of pacemaker or ICD	ICD	1339 (46%)	2900
	Pacemaker	1270 (44%)	
	ILR	291 (10%)	
*Rhythm*			
Sinus rhythm		67,799 (83%)	81,357
Atrial fibrillation/flutter		5072 (6%)	
Frequent ectopy		7846 (10%)	
Paced rhythm		640 (1%)	
*Medications*			
Aspirin		37,682 (44%)	85,641
Angiotensin-converting enzyme inhibitor		21,077 (24%)	87,821
Angiotensin receptor blocker		13,299 (16%)	83,119
Beta blocker		37,310 (43%)	86,767
Nitrate		6512 (8%)	81,400
Diuretic		24,586 (29%)	84,799
Statin		36,766 (43%)	85,502
*Contrast agent classification*			123,594
Linear (type I)		50,674 (41%)	
Macrocyclic (type II)		72,920 (59%)	
*Vital signs*			
Body surface area (kg/m^2^)		1.97 (1.78-2.15)	134,655
Systolic blood pressure (mmHg)		129 (117-143)	109,211
Diastolic blood pressure (mmHg)		74 (66-90)	109,183
Heart rate (bpm)		72 (63-83)	120,117
*Labs*			
Creatinine (ng/dL)		0.95 (0.80-1.17)	88,267
eGFR		79 (64-98)	74,059
*Outcomes*			
Mortality	Alive	123,017 (92%)	132,979
	Dead	9962 (8%)	

Data are presented as mean ± SD or median (IQR) or frequency (%) as appropriate. *AA* African American, *ARVC* arrhythmogenic right ventricular cardiomyopathy, *CAD* coronary artery disease, *DCM* dilated cardiomyopathy, e*GFR* estimated glomerular filtration rate, *HCM* hypertrophic cardiomyopathy, *ICD* implantable cardioverter defibrillator, *ILR* implantable loop recorder.

### 3.3 DICOM images

There were a total of 299,622,066 individual DICOM images in the SCMR Registry, representing approximately 100 terabytes of storage space usage. A code update was installed on the HeartIT server in 2016, allowing for the annotation of certain imaging sequences by name, specifically: cine, LGE (including slice orientation), and myocardial perfusion (rest and stress). Table 4 shows the number of patient exams that include at least one scan with sequence name annotation across the 77,871 scans performed since 2016. Short-axis cine represented the highest majority at 99% (76,859/77,871), with 66% (51,591/77,871) of exams including a short-axis LGE sequence, and 30% (23,241/77,871) having stress perfusion performed.

**Table 4:** Processed number of CMR exams after 2016 with tagged sequences.

Sequence	Number of exams (% total, 77,871)
Cine: LV 2 chamber	60,975 (78%)
Cine: LV 3 chamber	61,713 (79%)
Cine: LV 4 chamber	57,890 (74%)
Cine: LV short axis	76,859 (99%)
Perfusion: stress	23,241(30%)
Perfusion: rest	29,994 (39%)
LGE: LV 2 chamber	19,670 (25%)
LGE: LV 3 chamber	19,289 (25%)
LGE: LV 4 chamber	19,446 (25%)
LGE: LV short axis	51,591 (66%)

Data are presented as frequency (%) as appropriate. *LV* left ventricle*, LGE* late gadolinium enhancement.

### 3.4 CMR findings

Table 5 shows the CMR findings with corresponding completeness. The average left and right ventricular ejection fractions were 59% and 55%, respectively. Of the 24,153 stress CMR exams with reported findings, 70% (16,918/24,153) were normal, and 13% (3,231/24,153) reported a severe regional perfusion abnormality. With 85,316 exams (55% of the Registry) reporting LGE findings, 62% (53,032/85,316) showed no LGE, 18% (15,602/85,316) demonstrated non-ischemic pattern LGE, 17% (14,532/85,316) showed ischemic pattern LGE, and 3% (2,150/85,316) showed mixed LGE patterns.

**Table 5:** Cardiovascular magnetic resonance parameters within the Society for Cardiovascular Magnetic Resonance Registry.

Parameter		Value	Number of exams
LV end-diastolic volume (mL)		148 (189-117)	109,403
LV end-systolic volume (mL)		50 (41-90)	109,167
LV mass (gm)		129 (98-170)	65,835
LV end-diastolic dimension (cm)		5.1 (4.6-5.7)	125,422
LV ejection fraction (%)		59 (49-66%)	108,603
RV end-diastolic volume (mL)		146 (115-185)	83,542
RV end-systolic volume (mL)		65 (47-91)	83,356
RV ejection fraction (%)		55 (48-61%)	82,889
LVH	None	82,496 (78%)	106,194
	Mild	14,614 (14%)	
	Moderate	6031 (6%)	
	Severe	3053 (4%)	
RVH	Normal	92,235 (94%)	98,057
	Mild	3768 (4%)	
	Moderate	1599 (2%)	
	Severe	455 (0%)	
Wall motion	Normal	75,046 (66%)	113,089
	Mild-moderately hypokinetic	14,305 (13%)	
	Severely hypokinetic	8847 (8%)	
	Akinetic	10,889 (10%)	
	Dyskinetic	4002 (3%)	
Stress findings	Normal	16,918 (70%)	24,153
	Mildly abnormal	1694 (7%)	
	Moderately abnormal	2302 (10%)	
	Severely abnormal	3231 (13%)	
	Non-diagnostic	8 (0%)	
LGE pattern	None	53,032 (62%)	85,316
	Non-ischemic	15,602 (18%)	
	Ischemic	14,532 (17%)	
	Mixed ischemic and non-ischemic	2150 (3%)	

Data are presented as mean ± SD or frequency (%) as appropriate. *LGE* late gadolinium enhancement, *LV* left ventricle, *LVH* left ventricular hypertrophy, *RV* right ventricle, *RVH* right ventricular hypertrophy.

### 3.5 Follow-up and outcomes

**Fig. 5:**
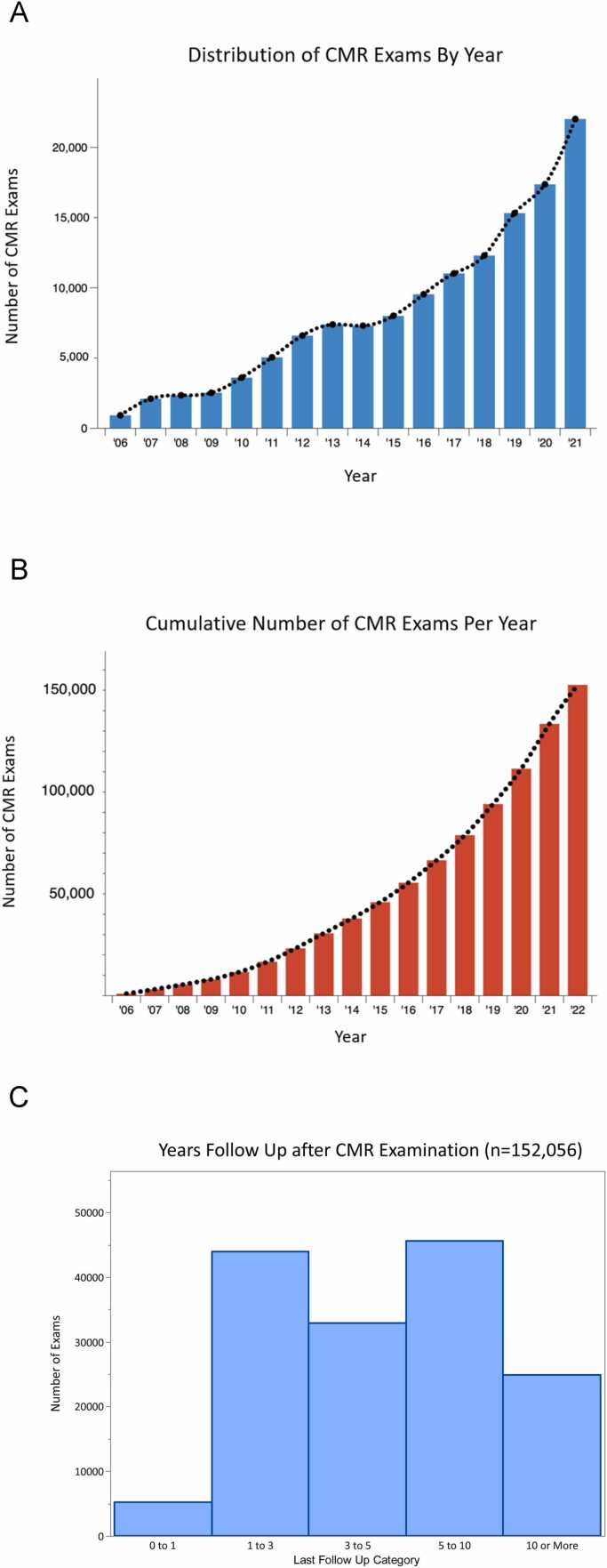
Distribution of CMR exams based on scan date (A), cumulative CMR exams with each year (B), and years of follow-up after scan (C). *CMR* cardiovascular magnetic resonance.

Fig. 5A and B shows the original scan date and cumulative scans, respectively, performed per year across all participating sites, demonstrating yearly SCMR Registry growth. Fig. 5C shows the years of available follow-up since the original scan. The median time elapsed since CMR was 3.6 years (IQR: 1.5-7 years). Approximately 29% (45,478/152,056) of CMR exams were performed 5-10 years ago and 16% (24,778/152,056) more than 10 years ago. This represents a potential of 456,678 patient-years of follow-up.

**Fig. 6:**
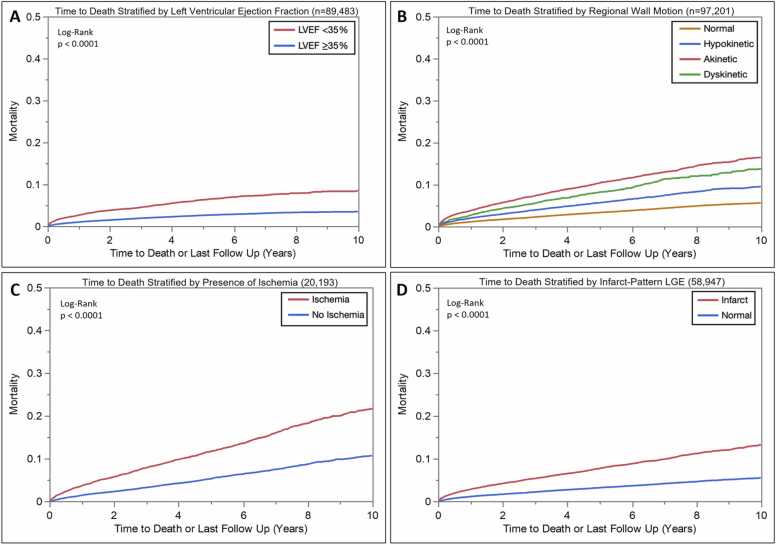
Mortality curves stratified by LVEF (A), regional wall motion (B), presence of inducible perfusion defects (C), and presence of infarct-pattern LGE (D). *LVEF* left ventricular ejection fraction, *LGE* late gadolinium enhancement.

The overall mortality rate was 8% (9,962/132,979) based on the most up-to-date records (Table 3). As an example of subgroup outcomes within the Registry, Fig. 6A-D shows mortality curves in reported LVEF, regional LV wall motion abnormalities, qualitative stress perfusion abnormalities, and the presence of infarct LGE. An LVEF <35% was associated with significantly increased mortality (chi-squared 452, log-rank p < 0.0001). The presence and severity of regional LV wall motion abnormalities were similarly associated with significantly increased mortality (chi-squared 1307, log-rank p < 0.0001). Compared to those with no stress perfusion abnormalities, the presence of stress-induced perfusion abnormalities was associated with significantly increased mortality (chi-squared 339, log-rank p < 0.0001). Lastly, compared to those with no LGE, the presence of infarct-pattern LGE was also associated with significantly increased mortality (chi-squared 626, p < 0.0001).

## 4 Discussion

The SCMR Registry represents the evolution from the initial GCMR registry established in 2014, to an expanding web-based, regulatory-compliant database, including DICOM images and searchable fields for research, education, and quality-control opportunities (Central Illustration). In 5 years since its creation, over 150,000 scans have been uploaded to the Registry, with an accelerating growth in site participation and ongoing investigations. The above results serve as examples to demonstrate the broad potential for future projects and are not intended to represent rigorous scientific investigation in specific disease cohorts.

RWE studies are complementary to RCTs in establishing clinical practice guidelines because they provide a broader and more representative view of diagnostic effectiveness in real-world settings. RCTs are considered the gold standard in clinical research because they are designed to control for bias and confounding factors. However, RCTs have limitations, such as limited generalizability and the inability to capture long-term outcomes. RWE studies, as demonstrated by previous SCMR Registry publications (Table 1), can help to identify real-world effectiveness, safety, and tolerability of non-invasive testing that may not be captured in RCTs. Large RWE datasets, such as the SCMR Registry, inherently do not control for biases or consistency but provide generalization and longer outcome data. Combining RCTs with RWE studies can provide a more comprehensive understanding of diagnostic effectiveness, leading to more robust clinical practice guidelines that are better suited toward personalized patient care. One example includes previous stress CMR Registry publications supporting a higher level of evidence for stress CMR utilization by the 2021 American College of Cardiology/American Heart Association (AHA) chest pain guidelines [5].

The roadmap from Registry data query to publication can be exemplified by the Heitner et al. study, which aimed to evaluate the prognostic value of vasodilator stress CMR in a large multicenter cohort of 9,151 patients with over 48,000 patient-years of follow-up [7]. The results showed that an abnormal vasodilator stress CMR was associated with a significantly higher risk of adverse cardiovascular events, including cardiovascular death, myocardial infarction, and coronary revascularization. Across the seven participating sites, the primary investigators coordinated with each site to gather the necessary data elements for analysis. Certain routine elements are more complete as shown in Tables 2 and 3, and thus readily available to extract with minimal effort. Less commonly reported elements, such as clinical risk factors, symptoms, medications, non-death MACE-related outcomes, AHA 17-segment-based wall motion, and stress perfusion, require active site participation to generate a complete dataset. The compilation of these efforts resulted in a publication demonstrating RWE risk stratification using stress CMR across multiple CAD subpopulations. Several other ongoing multicenter SCMR Registry Committee-approved projects include the investigation of sex-based LV remodeling differences in aortic regurgitation, evaluation of the prognostic implications of small myocardial infarcts in patients with normal contractile function, and a determination of clinical outcomes in patients with combined aortic regurgitation and myocardial scar [26].

Another key feature of the SCMR Registry is the inclusion of complete anonymized DICOM image sets with each exam. With nearly 300 million images, the Registry is a potential resource for academia-industry collaborations focused on developing, validating, and testing AI-powered tools, including automatic image analysis, reporting, and risk assessment. In addition to viewing the images, basic quantitative analysis, including cardiac chamber size, structure and function, tissue characterization, and strain, can be measured within the Registry platform. This allows Registry investigators to perform detailed multicenter quantitative measurements akin to a core lab. The large collection of DICOM images, paired with corresponding physician interpretation and quantitative reports within the Registry, provides a unique resource to develop and train ML- and AI-based algorithms. Current projects leveraging this feature include the development of a Tetralogy of Fallot biventricular shape atlas, implementation and validation of a cardiac amyloid neural network subtype prediction model, automated stress CMR analysis, and cardiac structure/function analysis.

Quality improvement is important across all imaging modalities for best practices, cost-effectiveness, and continued accreditation. The SCMR Registry includes International Classification of Disease codes, indications, sequences performed, and CPT codes, and could potentially serve as a hub to review exams for quality assurance. The ImageGuide Registry [27] is an example of how a registry can be successfully used for quality control. ImageGuide represents a joint collaborative effort between the American Society of Nuclear Cardiology and the American Society of Echocardiography, utilizing echocardiographic and nuclear imaging reports to support comparisons between local institutions and national aggregates. Another feature of ImageGuide is its recognition by the Centers for Medicare and Medicaid Services as a qualified clinical data registry, serving as a pathway for institutions to meet Merit-based Incentive Payment System requirements. These successes provide a roadmap toward streamlining accreditation reporting requirements, such as the Intersocietal Accreditation Commission, for quality assurance standards.

Lastly, the SCMR Registry potentially could serve as an educational tool to train both new and seasoned CMR readers, which represents a future direction of the Registry Committee. The linked DICOM images with clinical reports could be organized into a wide variety of case collections, ranging from stereotypical to complex cardiac diseases. Ongoing work is planned for structured access for educational purposes.

## 5 Limitations

As with any real-world data registry, there are a number of limitations and potential solutions. The data fields rely on the participating sites to populate them in a pre-specified manner, which may be absent if a report is generated using free text or dictation. However, the dataset can be updated post-hoc without amending the clinical report, usually when executing ongoing projects; thus, with the completion of each research project that adds information to the reports, the Registry data become more complete and thus more valuable for future investigations. The Registry predominantly contains CMR-related data with some cardiovascular history and hemodynamics, but other non-cardiac medical history and invasive or non-invasive test results are lacking. This requires active collaboration between the lead investigator with those at participating sites if additional data collection and non-routine image analysis are needed for a project. There is work ongoing to import clinical data from the electronic health record (EHR) into the Registry database, but currently, this must be done manually and often requires additional IRB approval. The mortality data field requires regular updating; however, this can be performed without IRB oversight, as mortality data are critical for local quality improvement efforts. Additionally, this Registry remains unique, as it provides access to both a searchable database of clinical parameters and corresponding DICOM images.

## 6 Conclusions and Future Directions

The SCMR Registry, following 5 years of growth, now includes a large cohort of over 150,000 scans with the primary mission to promote evidence-based utilization of CMR through a collaborative global effort to positively impact cardiovascular health outcomes. The Registry is unique in that it contains real-world CMR data with DICOM images, physician interpretation, quantitative results, and readily available outcome data. While current participating sites are predominantly based in the United States, there is one European participating site, demonstrating compliance with General Data Protection Regulation regulations. It remains a goal to expand Registry participation to other non-US sites, including locations in resource-limited settings to improve global collaboration and generalizability. Other future directions include refinement of educational tools, engagement of quality improvement and accreditation society metrics, and clinical EHR integration.

## Funding

O.P.S. is supported by The Robert F. Wolfe and Edgar T. Wolfe Foundation. Columbus, Ohio. B.S. is supported by NIH R01-HL153430. S.K. received funding from the DZHK (German Centre for Cardiovascular Research); and the BMBF (German Ministry of Education and Research) and is supported by the Deutsche Forschungsgemeinschaft (DFG, German Research Foundation) - SFB-1470 - B06 and received an unrestricted research grant from Philips Health Care, Germany. V.M.F. receives support from the National Institute for Health Research (NIHR), Oxford Biomedical Research Centre (BRC), the British Heart Foundation (BHF), and the British Heart Foundation Centre of Research Excellence, Oxford.

## Author contributions

**Steffen Petersen:** Writing – review and editing. **Venkateshwar Polsani:** Writing – review and editing. **Olga Toro-Salazar:** Writing – review and editing. **Kamran Shaikh:** Writing – review and editing. **Chetan Shenoy:** Writing – review and editing. **Monvadi B. Srichai:** Writing – review and editing. **Jeremy Slivnick:** Conceptualization, Data curation, Formal analysis, Investigation, Methodology, Software, Validation, Writing – review and editing. **John Grizzard:** Writing – review and editing. **Behzad Sharif:** Data curation, Formal analysis, Investigation, Visualization, Writing – review and editing. **Sebastian Kelle:** Writing – review and editing. **Matthew S. Tong:** Conceptualization, Data curation, Formal analysis, Investigation, Methodology, Project administration, Resources, Supervision, Validation, Writing – original draft, Writing – review and editing, Software, Visualization. **Simon Lee:** Writing – review and editing. **Orlando Simonetti:** Conceptualization, Data curation, Formal analysis, Investigation, Methodology, Project administration, Resources, Supervision, Validation, Visualization, Writing – original draft, Writing – review and editing. **Maan Malahfji:** Writing – review and editing. **W. Benjamin Wince:** Writing – review and editing. **Kanae Mukai:** Investigation, Validation, Visualization, Writing – review and editing. **Priya Chudgar:** Writing – review and editing. **Afshin Farzaneh-Far:** Conceptualization, Methodology, Writing – review and editing. **Matthew Judd:** Data curation, Formal analysis, Methodology, Software. **Sadeer Al-Kindi:** Writing – review and editing. **Robert Judd:** Conceptualization, Data curation, Formal analysis, Investigation, Methodology, Resources, Software, Supervision, Validation, Visualization, Writing – review and editing. **Angel Chan:** Writing – review and editing. **Dipan Shah:** Methodology, Project administration, Supervision, Writing – review and editing. **George Dibu:** Writing – review and editing. **Michael Elliot:** Writing – review and editing. **Vanessa Ferreira:** Supervision, Writing – review and editing. **Jadranka Stojanovska:** Writing – review and editing. **Qian Tao:** Writing – review and editing. **Han Kim:** Conceptualization, Investigation, Methodology, Writing – review and editing. **Janet Wei:** Writing – review and editing. **Alistair Young:** Conceptualization, Investigation, Supervision, Writing – review and editing. **Jonathan Weinsaft:** Writing – review and editing. **Lilia Sierra-Galan:** Investigation, Methodology, Supervision, Writing – review and editing.

## Ethics approval and consent

Participating sites have obtained either approval or waiver from an ethics or regulatory board before submitting data to the SCMR Registry.

## Consent for publication

Not applicable.

## Declaration of competing interests

The authors declare that they have no known competing financial interests or personal relationships that could have appeared to influence the work reported in this paper.

## Data Availability

The datasets used and/or analyzed during the current study are available from the corresponding author upon reasonable request.
