# Supplementary material for: The Society for Cardiovascular Magnetic Resonance Registry at 150,000
Source: J Cardiovasc Magn Reson. 2024 Jul 4;26(2):101055. doi: 10.1016/j.jocmr.2024.101055 (PMC11314894; doi:10.1016/j.jocmr.2024.101055)
Supplement: Supplementary file 1 — Supplementary material. [file mmc1.docx]

Supplemental Table: All available data fields within the SCMR Registry

| **Data Field Name** | **Data Field Location** |
| --- | --- |
| Gender | Scan Info - General |
| Race | Scan Info - General |
| Age | Scan Info - General |
| Vital Status | Scan Info - General |
| Death Date (Time-Shifted) | Scan Info - General |
| Death Source | Scan Info - General |
| Study Date (Time-Shifted) | Scan Info - General |
| Visual Ejection Fraction | Core Exam - Measurements - Volumetric Analysis |
| Stress Protocol | Stress - Stress Protocol |
| Peak Rate | Stress - Stress Protocol - Dobutamine |
| Atropine Dose | Stress - Stress Protocol - Dobutamine |
| Amount | Stress - Stress Protocol - Adenosine |
| Vial Size | Stress - Stress Protocol - Adenosine |
| Infusion Time | Stress - Stress Protocol - Adenosine |
| Amount | Stress - Stress Protocol - Regadenoson |
| Systolic BP | Stress - Resting Data |
| Diastolic BP | Stress - Resting Data |
| Resting HR | Stress - Resting Data |
| Medications Taken Today | Stress - Resting Data |
| ECG | Stress - Resting Data |
| Peak Systolic BP | Stress - Stress Data |
| Peak Diastolic BP | Stress - Stress Data |
| Peak HR | Stress - Stress Data |
| Reason for Termination | Stress - Stress Data |
| Complications | Stress - Stress Data |
| Post Stress ECG | Stress - Stress Data |
| End Diastolic Volume | Core Exam - Measurements - Volumetric Analysis - LV |
| End Systolic Volume | Core Exam - Measurements - Volumetric Analysis - LV |
| Cardiac Output | Core Exam - Measurements - Volumetric Analysis - LV |
| Myocardial Mass | Core Exam - Measurements - Volumetric Analysis - LV |
| Stroke Volume | Core Exam - Measurements - Volumetric Analysis - LV |
| Ejection Fraction | Core Exam - Measurements - Volumetric Analysis - LV |
| End Diastolic Volume | Core Exam - Measurements - Volumetric Analysis - LV - Normalized |
| End Systolic Volume | Core Exam - Measurements - Volumetric Analysis - LV - Normalized |
| Cardiac Output | Core Exam - Measurements - Volumetric Analysis - LV - Normalized |
| Myocardial Mass | Core Exam - Measurements - Volumetric Analysis - LV - Normalized |
| Stroke Volume | Core Exam - Measurements - Volumetric Analysis - LV - Normalized |
| Diagnosis | Congenital - Diagnosis |
| Repair | Congenital - Repair |
| Systemic Venous Anatomy | Congenital - Morphology - Morphology |
| Pulmonary Venous Connections | Congenital - Morphology - Morphology |
| Atrial Septum | Congenital - Morphology - Morphology |
| Direct Shunt Flow | Congenital - Morphology - Atrial Septum |
| Ventricular Septum | Congenital - Morphology - Morphology |
| Direct Shunt Flow | Congenital - Morphology - Ventricular Septum |
| Conotruncal Anatomy | Congenital - Morphology - Morphology |
| Systemic Venous Connections | Congenital - Morphology - Morphology |
| Atrial Septum | Congenital - Morphology - Morphology |
| Ventricular Septum | Congenital - Morphology - Morphology |
| Conotruncal Anatomy | Congenital - Morphology - Morphology |
| Sidedness | Congenital - Morphology - Aortic Arch |
| Branching | Congenital - Morphology - Aortic Arch |
| Coarctation | Congenital - Morphology - Aortic Arch |
| PDA | Congenital - Morphology - Aortic Arch |
| Main PA | Congenital - Morphology - Pulmonary Arteries |
| Main PA Diameter | Congenital - Morphology - Pulmonary Arteries |
| Right PA | Congenital - Morphology - Pulmonary Arteries |
| Right PA Diameter | Congenital - Morphology - Pulmonary Arteries |
| Left PA | Congenital - Morphology - Pulmonary Arteries |
| Left PA Diameter | Congenital - Morphology - Pulmonary Arteries |
| Sedation Used? | Scan Info - General - Sedation |
| Type | Scan Info - General - Sedation |
| Dose | Scan Info - General - Sedation |
| Any Reaction? | Scan Info - General - Sedation |
| Type | Scan Info - General - Contrast Agent |
| Expiration Date | Scan Info - Contrast Agent - Expiration Date |
| Gadolinium Concentration | Scan Info - General - Contrast Agent |
| Volume Administered | Scan Info - General - Contrast Agent |
| Amount Administered | Scan Info - General - Contrast Agent |
| Dosage | Scan Info - General - Contrast Agent |
| Serum Creatinine | Scan Info - General - Contrast Agent |
| GFR | Scan Info - General - Contrast Agent |
| Creatinine Date | Scan Info - General - Contrast Agent |
| Type | Scan Info - General - Medication Administered During Scan |
| Other(mg), Total Dose | Scan Info - General - Medication Administered During Scan |
| Pulse Sequences | Scan Info - General - Pulse Sequences |
| End Diastolic Volume | Core Exam - Measurements - Volumetric Analysis - RV |
| End Systolic Volume | Core Exam - Measurements - Volumetric Analysis - RV |
| Cardiac Output | Core Exam - Measurements - Volumetric Analysis - RV |
| Myocardial Mass | Core Exam - Measurements - Volumetric Analysis - RV |
| Stroke Volume | Core Exam - Measurements - Volumetric Analysis - RV |
| Ejection Fraction | Core Exam - Measurements - Volumetric Analysis - RV |
| End Diastolic Volume | Core Exam - Measurements - Volumetric Analysis - RV - Normalized |
| End Systolic Volume | Core Exam - Measurements - Volumetric Analysis - RV - Normalized |
| Cardiac Output | Core Exam - Measurements - Volumetric Analysis - RV - Normalized |
| Stroke Volume | Core Exam - Measurements - Volumetric Analysis - RV - Normalized |
| Cardiac Output HR | Core Exam - Measurements - Volumetric Analysis |
| History of Hypertension | Medical History - Risk Factors |
| History of Hyperlipidemia | Medical History - Risk Factors |
| Family History of CAD | Medical History - Risk Factors |
| Menopause | Medical History - Risk Factors |
| History of Diabetes | Medical History - Risk Factors |
| Type | Medical History - Risk Factors - History of Diabetes |
| History of Smoking | Medical History - Risk Factors |
| Family History of SCD | Medical History - Risk Factors |
| Wall Thickness - Anteroseptal | Core Exam - Measurements - LV Dimensions |
| Wall Thickness - Inferolateral | Core Exam - Measurements - LV Dimensions |
| Wall Thickness - Maximum | Core Exam - Measurements - LV Dimensions |
| LV EDD | Core Exam - Measurements - LV Dimensions |
| LV ESD | Core Exam - Measurements - LV Dimensions |
| Coronary Artery Disease (prior to MI, if any) | Medical History - Cardiac History |
| Myocardial Infarction | Medical History - Cardiac History |
| Moderate/Severe Valvular Disease | Medical History - Cardiac History |
| Type(s) | Medical History - Cardiac History - Moderate/Severe Valvular Disease |
| Prior Repair(s)/Replacement(s) | Medical History - Cardiac History - Moderate/Severe Valvular Disease |
| History of Peripheral Artery Disease | Medical History - Cardiac History |
| Congenital Heart Disease | Medical History - Cardiac History |
| Non-ischemic Cardiomyopathy | Medical History - Cardiac History |
| Type | Medical History - Cardiac History - Non-ischemic Cardiomyopathy |
| Cardiac Device | Medical History - Cardiac History |
| Device Make | Medical History - Cardiac History |
| Atrial Fibrillation | Medical History - Cardiac History |
| Type | Medical History - Cardiac History - Atrial Fibrillation |
| Wall Thickness | Core Exam - Anatomy - LV |
| LVH Pattern | Core Exam - Anatomy - LV |
| Cavity Size | Core Exam - Anatomy - LV |
| Contractility | Core Exam - Anatomy - LV |
| Mass/Thrombus | Core Exam - Anatomy - LV |
| Wall Thickness | Core Exam - Anatomy - RV |
| Cavity Size | Core Exam - Anatomy - RV |
| Contractility | Core Exam - Anatomy - RV |
| Mass/Thrombus | Core Exam - Anatomy - RV |
| RV Infiltration | Core Exam - Anatomy - Right Ventricle |
| Pacemaker/Defibrillator Wire | Core Exam - Anatomy - RV |
| Normal | Description - Ventricular Septum |
| Muscular VSD (location) | Description - Ventricular Septum |
| Muscular VSD (size) | Description - Ventricular Septum |
| Muscular VSD Measurement | Description - Ventricular Septum |
| Membranous / Perimembranous / Paramembranous / Conoventricular VSD | Description - Ventricular Septum |
| Ventricular septal geometry | Description - Ventricular Septum |
| Normal | Description - Atrial Septum |
| Secundum ASD | Description - Atrial Septum |
| Shunting | Description - Atrial Septum |
| Cavity Size | Core Exam - Anatomy - LA |
| Mass/Thrombus | Core Exam - Anatomy - LA |
| Cavity Size | Core Exam - Anatomy - RA |
| Mass/Thrombus | Core Exam - Anatomy - RA |
| Additional Findings | Core Exam - Anatomy - RA |
| Pacemaker/Defibrillator Wire | Core Exam - Anatomy - RA |
| Pericardium | Core Exam - Anatomy - Pericardium |
| Thickness | Core Exam - Anatomy - Pericardium - Pericardium |
| Effusion | Core Exam - Anatomy - Pericardium |
| Maximum Dimension | Core Exam - Anatomy - Pericardium - Effusion |
| Location | Core Exam - Anatomy - Pericardium |
| Collapse | Core Exam - Anatomy - Pericardium |
| Pleural Effusion | Core Exam - Anatomy - Pleural Effusion |
| Chest Pain | Medical History - Symptoms |
| Type | Medical History - Symptoms - Chest Pain |
| Dyspnea | Medical History - Symptoms |
| Aortic Valve Annulus | Description - Aortic Valve |
| Aortic Valve Leaflets | Core Exam - Valves - Aortic |
| Aortic Morphology | Core Exam - Valves - Aortic Morphology |
| Aortic Mass/Thrombus | Core Exam - Valves - Aortic |
| Prosthetic Aortic Valve | Core Exam - Valves - Aortic |
| Aortic Regurgitation | Core Exam - Valves - Aortic |
| Aortic Regurgitant Volume | Core Exam - Valves - Aortic |
| Aortic Regurgitant Fraction | Core Exam - Valves - Aortic |
| Aortic Stenosis | Core Exam - Valves - Aortic |
| Planimetered Aortic Valve Area | Core Exam - Valves - Aortic |
| Peak Aortic Valve Velocity | Core Exam - Valves - Aortic |
| Peak Aortic Valve Gradient | Core Exam - Valves - Aortic |
| Mitral Valve Annulus | Description - Mitral Valve |
| Mitral Valve Leaflets | Core Exam - Valves - Mitral |
| Mitral Morphology | Core Exam - Valves - Mitral |
| Mitral Mass/Thrombus | Core Exam - Valves - Mitral |
| Prosthetic Mitral Valve | Core Exam - Valves - Mitral |
| MVP | Core Exam - Valves - Mitral |
| MVP Severity | Core Exam - Valves - Mitral |
| Mitral Regurgitation | Core Exam - Valves - Mitral |
| Mitral Regurgitant Volume | Core Exam - Valves - Mitral |
| Mitral Regurgitant Fraction | Core Exam - Valves - Mitral |
| Mitral Stenosis | Core Exam - Valves - Mitral |
| Peak Mitral Valve Velocity | Core Exam - Valves - Mitral |
| Peak Mitral Valve Gradient | Core Exam - Valves - Mitral |
| Tricuspid Valve Annulus | Description - Tricuspid Valve |
| Tricuspid Valve Leaflets | Core Exam - Valves - Tricuspid |
| Tricuspid Morphology | Core Exam - Valves - Tricuspid |
| Tricuspid Mass/Thrombus | Core Exam - Valves - Tricuspid |
| Prosthetic Tricuspid Valve | Core Exam - Valves - Tricuspid |
| Tricuspid Regurgitation | Core Exam - Valves - Tricuspid |
| Tricuspid Regurgitant Volume | Core Exam - Valves - Tricuspid |
| Tricuspid Regurgitant Fraction | Core Exam - Valves - Tricuspid |
| Tricuspid Stenosis | Core Exam - Valves - Tricuspid |
| Peak Tricuspid Valve Velocity | Core Exam - Valves - Tricuspid |
| Peak Tricuspid Valve Gradient | Core Exam - Valves - Tricuspid |
| Pulmonic Valve Annulus | Description - Pulmonic Valve |
| Pulmonic Valve Leaflets | Core Exam - Valves - Pulmonic |
| Pulmonic Morphology | Core Exam - Valves - Pulmonic |
| Pulmonic Mass/Thrombus | Core Exam - Valves - Pulmonic |
| Prosthetic Pulmonic Valve | Core Exam - Valves - Pulmonic |
| Pulmonic Regurgitation | Core Exam - Valves - Pulmonic |
| Pulmonic Regurgitant Volume | Core Exam - Valves - Pulmonic |
| Pulmonic Regurgitant Fraction | Core Exam - Valves - Pulmonic |
| Pulmonic Stenosis | Core Exam - Valves - Pulmonic |
| Planimetered Pulmonic Valve Area | Core Exam - Valves - Pulmonic |
| Peak Pulmonic Valve Velocity | Core Exam - Valves - Pulmonic |
| Peak Pulmonic Valve Gradient | Core Exam - Valves - Pulmonic |
| Neo-pulmonic Regurgitation | Description - Congenital - Pulmonic Valve |
| Neo-pulmonic Regurgitant Fraction | Description - Congenital - Pulmonic Valve |
| Neo-pulmonic Stenosis | Description - Congenital - Pulmonic Valve |
| Aortic Root Size | Core Exam - Measurements - Aortic Root Dimensions |
| Anatomy | Description - Coronaries |
| Heart Failure | Medical History - Heart Failure |
| NYHA Class | Medical History - Heart Failure |
| HF Stage | Medical History - Heart Failure |
| VSD patch repair | Description - Congenital - Repair |
| VSD suture repair | Description - Congenital - Repair |
| ASD patch repair | Description - Congenital - Repair |
| ASD suture closure | Description - Congenital - Repair |
| PFO suture closure | Description - Congenital - Repair |
| Patent ductus arteriosus closure | Description - Congenital - Repair |
| Tetralogy of Fallot repair | Description - Congenital - Repair |
| Aortico-pulmonary shunt | Description - Congenital - Repair |
| Pulmonary valvuloplasty | Description - Congenital - Repair |
| Pulmonary valve replacement | Description - Congenital - Repair |
| Pulmonary valve replacement: transcatheter | Description - Congenital - Repair |
| Relief of subpulmonary stenosis | Description - Congenital - Repair |
| Pulmonary arterioplasty | Description - Congenital - Repair |
| Stent angioplasty of pulmonary artery | Description - Congenital - Repair |
| Coarctation of the Aorta repair | Description - Congenital - Repair |
| Stent angioplasty of the SVC | Description - Congenital - Repair |
| Position | Description - Congenital |
| Visceral Situs | Description - Congenital |
| Atrial Situs | Description - Congenital |
| ACE-I | Medical History - Medications |
| ASA | Medical History - Medications |
| Coumadin | Medical History - Medications |
| Novel Oral Anticoagulation | Medical History - Medications |
| Type(s) | Medical History - Medications - Novel Oral Anticoagulation |
| Beta Blocker | Medical History - Medications |
| Nitrate | Medical History - Medications |
| Diuretic | Medical History - Medications |
| Insulin | Medical History - Medications |
| Spironolactone | Medical History - Medications |
| Other Antiarrhythmic | Medical History - Medications |
| ARB | Medical History - Medications |
| Thienopyridine/CPTP Antiplatelet Agents: prasugrel (Effient), ticlopidine (Ticlid), clopidogrel (Plavix), ticagrelor (Brilinta) | Medical History - Medications |
| Heparin | Medical History - Medications |
| Calcium Blocker | Medical History - Medications |
| Digoxin | Medical History - Medications |
| Hormone Therapy | Medical History - Medications |
| Oral Antidiabetic | Medical History - Medications |
| Statin | Medical History - Medications |
| Amiodarone | Medical History - Medications |
| Ventricular looping | Description - Congenital |
| Great Artery Orientation | Description - Congenital |
| Systemic Venous Anatomy | Description - Congenital |
| Right SVC | Description - Congenital - Systemic venous anatomy |
| Left SVC | Description - Congenital - Systemic venous anatomy |
| Innominate/bridging vein | Description - Congenital - Systemic venous anatomy |
| Inferior vena cava | Description - Congenital - Systemic venous anatomy |
| Surgical SVC to PA anastomosis | Description - Congenital - Systemic venous anatomy |
| Pulmonary Venous Anatomy | Description - Congenital |
| Partial anomalous pulmonary venous return | Description - Congenital - Pulmonary Venous Anatomy |
| Tetralogy of Fallot | Description - Conotruncal Anatomy |
| Right-sided aortic arch | Description - Aortic Arch |
| Double aortic arch | Description - Aortic Arch |
| Coarctation severity | Congenital - Morphology - Aortic Arch - Coarctation |
| Forward flow in diastole | Congenital - Morphology - Aortic Arch |
| Subaortic stenosis type | Description - Outflow Tract |
| Subaortic stenosis severity | Description - Outflow Tract |
| Supravalvar aortic stenosis severity | Description - Outflow Tract |
| Subpulmonary stenosis type | Description - Outflow Tract |
| Subpulmonary stenosis severity | Description - Outflow Tract |
| Supravalvar pulmonary stenosis severity | Description - Outflow Tract |
| Laboratory Tests | Medical History - Laboratory Tests |
| LDL Date | Medical History - Laboratory Tests - LDL |
| LDL Value | Medical History - Laboratory Tests - LDL |
| LDL ULN | Medical History - Laboratory Tests - LDL |
| Cholesterol Date | Medical History - Laboratory Tests - Cholesterol |
| Cholesterol Value | Medical History - Laboratory Tests - Cholesterol |
| Cholesterol ULN | Medical History - Laboratory Tests - Cholesterol |
| HDL Date | Medical History - Laboratory Tests - HDL |
| HDL Value | Medical History - Laboratory Tests - HDL |
| HDL ULN | Medical History - Laboratory Tests - HDL |
| Triglycerides Date | Medical History - Laboratory Tests - Triglycerides |
| Triglycerides Value | Medical History - Laboratory Tests - Triglycerides |
| Triglycerides ULN | Medical History - Laboratory Tests - Triglycerides |
| HbA1c Date | Medical History - Laboratory Tests - HbA1c |
| HbA1c Value | Medical History - Laboratory Tests - HbA1c |
| BNP Date | Medical History - Laboratory Tests - BNP |
| BNP Value | Medical History - Laboratory Tests - BNP |
| BNP ULN | Medical History - Laboratory Tests - BNP |
| Scan Type | Scan Info - General - Setup |
| Patient Type | Scan Info - General - Setup |
| Incomplete Scan | Scan Info - General - Setup |
| Reason(s) for Scan | Scan Info - General - Setup |
| CPT Codes | Scan Info - Billing |
| HCPCS Codes | Scan Info - Billing |
| ICD10 Codes | Scan Info - Billing |
| Base Anterior Wall Motion | 17 Segment (LV1) |
| Base Anteroseptal Wall Motion | 17 Segment (LV2) |
| Base Inferoseptal Wall Motion | 17 Segment (LV3) |
| Base Inferior Wall Motion | 17 Segment (LV4) |
| Base Inferolateral Wall Motion | 17 Segment (LV5) |
| Base Anterolateral Wall Motion | 17 Segment (LV6) |
| Mid Anterior Wall Motion | 17 Segment (LV7) |
| Mid Anteroseptal Wall Motion | 17 Segment (LV8) |
| Mid Inferoseptal Wall Motion | 17 Segment (LV9) |
| Mid Inferior Wall Motion | 17 Segment (LV10) |
| Mid Inferolateral Wall Motion | 17 Segment (LV11) |
| Mid Anterolateral Wall Motion | 17 Segment (LV12) |
| Apical Anterior Wall Motion | 17 Segment (LV13) |
| Apical Septal Wall Motion | 17 Segment (LV14) |
| Apical Inferior Wall Motion | 17 Segment (LV15) |
| Apical Lateral Wall Motion | 17 Segment (LV16) |
| Apex Wall Motion | 17 Segment (LV17) |
| RV Basal Anterior Wall Motion | 17 Segment (RV1) |
| RV Basal Inferior Wall Motion | 17 Segment (RV2) |
| RV Mid Wall Motion | 17 Segment (RV3) |
| RV Apical Wall Motion | 17 Segment (RV4) |
| Diameter | Core Exam - Measurements - LA Dimensions LV Systole |
| Area - 2 Chamber | Core Exam - Measurements - LA Dimensions LV Systole |
| Length - 2 Chamber | Core Exam - Measurements - LA Dimensions LV Systole |
| Area - 4 Chamber | Core Exam - Measurements - LA Dimensions LV Systole |
| Length - 4 Chamber | Core Exam - Measurements - LA Dimensions LV Systole |
| Volume | Core Exam - Measurements - LA Dimensions LV Systole |
| Volume Normalized | Core Exam - Measurements - LA Dimensions LV Systole |
| Base Anterior Hyperenhancement | 17 Segment (LV1) |
| Base Anteroseptal Hyperenhancement | 17 Segment (LV2) |
| Base Inferoseptal Hyperenhancement | 17 Segment (LV3) |
| Base Inferior Hyperenhancement | 17 Segment (LV4) |
| Base Inferolateral Hyperenhancement | 17 Segment (LV5) |
| Base Anterolateral Hyperenhancement | 17 Segment (LV6) |
| Mid Anterior Hyperenhancement | 17 Segment (LV7) |
| Mid Anteroseptal Hyperenhancement | 17 Segment (LV8) |
| Mid Inferoseptal Hyperenhancement | 17 Segment (LV9) |
| Mid Inferior Hyperenhancement | 17 Segment (LV10) |
| Mid Inferolateral Hyperenhancement | 17 Segment (LV11) |
| Mid Anterolateral Hyperenhancement | 17 Segment (LV12) |
| Apical Anterior Hyperenhancement | 17 Segment (LV13) |
| Apical Septal Hyperenhancement | 17 Segment (LV14) |
| Apical Inferior Hyperenhancement | 17 Segment (LV15) |
| Apical Lateral Hyperenhancement | 17 Segment (LV16) |
| Apex Hyperenhancement | 17 Segment (LV17) |
| RV Basal Anterior Hyperenhancement | 17 Segment (RV1) |
| RV Basal Inferior Hyperenhancement | 17 Segment (RV2) |
| RV Mid Hyperenhancement | 17 Segment (RV3) |
| RV Apical Hyperenhancement | 17 Segment (RV4) |
| Height | Scan Info - General - Vitals |
| Height | Scan Info - General - Vitals |
| Weight | Scan Info - General - Vitals |
| Weight | Scan Info - General - Vitals |
| BSA | Scan Info - General - Vitals |
| Systolic BP | Scan Info - General - Vitals |
| Diastolic BP | Scan Info - General - Vitals |
| Baseline HR | Scan Info - General - Vitals |
| Heart Rhythm | Scan Info - General - Vitals |
| Common Dimensions (2) | Pulmonary Vein - Left Side Measurements |
| Upper Dimensions (2) | Pulmonary Vein - Right Side Measurements |
| Lower Dimensions (2) | Pulmonary Vein - Right Side Measurements |
| Base Anterior Stress Perfusion | 17 Segment (LV1) |
| Base Anteroseptal Stress Perfusion | 17 Segment (LV2) |
| Base Inferoseptal Stress Perfusion | 17 Segment (LV3) |
| Base Inferior Stress Perfusion | 17 Segment (LV4) |
| Base Inferolateral Stress Perfusion | 17 Segment (LV5) |
| Base Anterolateral Stress Perfusion | 17 Segment (LV6) |
| Mid Anterior Stress Perfusion | 17 Segment (LV7) |
| Mid Anteroseptal Stress Perfusion | 17 Segment (LV8) |
| Mid Inferoseptal Stress Perfusion | 17 Segment (LV9) |
| Mid Inferior Stress Perfusion | 17 Segment (LV10) |
| Mid Inferolateral Stress Perfusion | 17 Segment (LV11) |
| Mid Anterolateral Stress Perfusion | 17 Segment (LV12) |
| Apical Anterior Stress Perfusion | 17 Segment (LV13) |
| Apical Septal Stress Perfusion | 17 Segment (LV14) |
| Apical Inferior Stress Perfusion | 17 Segment (LV15) |
| Apical Lateral Stress Perfusion | 17 Segment (LV16) |
| Apex Stress Perfusion | 17 Segment (LV17) |
| Base Anterior Interpretation | 17 Segment (LV1) |
| Base Anteroseptal Interpretation | 17 Segment (LV2) |
| Base Inferoseptal Interpretation | 17 Segment (LV3) |
| Base Inferior Interpretation | 17 Segment (LV4) |
| Base Inferolateral Interpretation | 17 Segment (LV5) |
| Base Anterolateral Interpretation | 17 Segment (LV6) |
| Mid Anterior Interpretation | 17 Segment (LV7) |
| Mid Anteroseptal Interpretation | 17 Segment (LV8) |
| Mid Inferoseptal Interpretation | 17 Segment (LV9) |
| Mid Inferior Interpretation | 17 Segment (LV10) |
| Mid Inferolateral Interpretation | 17 Segment (LV11) |
| Mid Anterolateral Interpretation | 17 Segment (LV12) |
| Apical Anterior Interpretation | 17 Segment (LV13) |
| Apical Septal Interpretation | 17 Segment (LV14) |
| Apical Inferior Interpretation | 17 Segment (LV15) |
| Apical Lateral Interpretation | 17 Segment (LV16) |
| Apex Interpretation | 17 Segment (LV17) |
| RV Basal Anterior Interpretation | 17 Segment (RV1) |
| RV Basal Inferior Interpretation | 17 Segment (RV2) |
| RV Mid Interpretation | 17 Segment (RV3) |
| RV Apical Interpretation | 17 Segment (RV4) |
| LV Scar Size (17 segment) | 17 Segment Interpretation |
| Hematocrit | Scan Info - Lab Result |
| Hematocrit Date | Scan Info - Lab Result |
| Pre-contrast T1 LV Myocardium | Core Exam - Measurements - Extracellular Volume Measurement |
| Pre-contrast T1 LV Cavity | Core Exam - Measurements - Extracellular Volume Measurement |
| Post-contrast T1 LV Myocardium | Core Exam - Measurements - Extracellular Volume Measurement |
| Post-contrast T1 LV Cavity | Core Exam - Measurements - Extracellular Volume Measurement |
| ECV | Core Exam - Measurements - Extracellular Volume Measurement |
| Annulus | Core Exam - Measurements - Aortic Root Dimensions |
| Sinus of Valsalva | Core Exam - Measurements - Aortic Root Dimensions |
| Sinotubular Junction | Core Exam - Measurements - Aortic Root Dimensions |
| Diameter | Core Exam - Measurements - RA Dimensions RV Systole |
| Area - 4 Chamber | Core Exam - Measurements - RA Dimensions RV Systole |
| Length - 4 Chamber | Core Exam - Measurements - RA Dimensions RV Systole |
| Annulus Z-score | Core Exam - Measurements - Z-Scores - Aortic |
| Sinus of Valsalva Z-score | Core Exam - Measurements - Z-Scores - Aortic |
| Sinotubular Junction Z-score | Core Exam - Measurements - Z-Scores - Aortic |
| Proximal to the origin of the brachiocephalic | Core Exam - Measurements - Z-Scores - Aortic |
| Proximal to the origin of the brachiocephalic Z-score | Core Exam - Measurements - Z-Scores - Aortic |
| First transverse segment | Core Exam - Measurements - Z-Scores - Aortic |
| First transverse segment Z-score | Core Exam - Measurements - Z-Scores - Aortic |
| Second transverse segment | Core Exam - Measurements - Z-Scores - Aortic |
| Second transverse segment Z-score | Core Exam - Measurements - Z-Scores - Aortic |
| Isthmic region | Core Exam - Measurements - Z-Scores - Aortic |
| Isthmic region Z-score | Core Exam - Measurements - Z-Scores - Aortic |
| Descending aorta | Core Exam - Measurements - Z-Scores - Aortic |
| Descending aorta Z-score | Core Exam - Measurements - Z-Scores - Aortic |
| Thoracoabdominal aorta at the level of the diaphragm | Core Exam - Measurements - Z-Scores - Aortic |
| Thoracoabdominal aorta at the level of the diaphragm Z-score | Core Exam - Measurements - Z-Scores - Aortic |
| Myocardial T2* | Core Exam - Measurements - Iron Quantification |
| Liver T2* | Core Exam - Measurements - Iron Quantification |
| Qp | Core Exam - Measurements - Flow Analysis |
| Qs | Core Exam - Measurements - Flow Analysis |
| Qp/Qs | Core Exam - Measurements - Flow Analysis |
| Visceral Situs | Congenital - Morphology - Morphology |
| Atrial Situs | Congenital - Morphology - Morphology |
| Mass/Thrombus Size | Core Exam - Anatomy - LV |
| Mass/Thrombus Size | Core Exam - Anatomy - RV |
| Ventricular Septum | Core Exam - Anatomy - Ventricular Septum |
| Atrial Septum | Core Exam - Anatomy - Atrial Septum |
| ICD9 Codes | Scan Info - Billing |
| Ejection Fraction | 17 Segment Interpretation |
| Scanner Strength | Scan Info - General - Setup |
| Study Quality | Scan Info - General - Setup |
| Critical Result | Summary |
| Appropriateness of Order | Billing |
| RV EDD | Core Exam - Measurements - LV/RV Dimensions |
| RV ESD | Core Exam - Measurements - LV/RV Dimensions |
| RA Area - 4 Chamber | Core Exam - Measurements - LA (Systole) / RA Dimensions |
| HR | Stress Stages (1) |
| HR | Stress Stages (3) |
| HR | Stress Stages (4) |
| HR | Stress Stages (5) |
| HR | Stress Stages (6) |
| RA Diameter | Core Exam - Measurements - LA (Systole) / RA Dimensions |
| HR | Stress Stages (7) |
| HR | Stress Stages (8) |
| Clinical Registry | Scan Info - General - Setup |

ACE-I, angiotensin converting enzyme inhibitor; ARB, Angiotensin Receptor Blocker; ASA, aspirin; ASD, atrial septal defect; BNP, brain natriuretic peptide; BP, blood pressure; BSA, body surface area; CAD, coronary artery disease; CPTP, cyclopentyltriazolopyrimidine; ECG, electrocardiogram; ECV, extracellular volume; EDD, end diastolic diameter; ESD, end systolic diameter; GFR, glomerular filtration rate; HbA1c, hemoglobin A1c; HCPCS, Healthcare Common Procedure Coding System; HDL, high-density lipoprotein; HF, heart failure; HR, heart rate; LA, left atrium; LDL, low-density lipoprotein; LV, left ventricle; LVH, left ventricular hypertrophy; MI, myocardial infarction; MVP, mitral valve prolapse; NYHA, New York Heart Association; PDA, patent ductus arteriosus; PA, pulmonary artery; PFO, patent foramen ovale; RA, right atrium; RV, right ventricle; RVH, right ventricular hypertrophy; SCD, sudden cardiac death; SVC, superior vena cava; ULN, upper limit normal; VSD, ventricular septal defect.
